# Healthcare Utilization and Clinical Outcomes after Catheter Ablation of Atrial Flutter

**DOI:** 10.1371/journal.pone.0100509

**Published:** 2014-07-01

**Authors:** Thomas A. Dewland, David V. Glidden, Gregory M. Marcus

**Affiliations:** 1 Department of Internal Medicine, Division of Cardiology, Electrophysiology Section, University of California San Francisco, San Francisco, California, United States of America; 2 Department of Epidemiology and Biostatistics, University of California San Francisco, San Francisco, California, United States of America; University of Illinois at Chicago, United States of America

## Abstract

Atrial flutter ablation is associated with a high rate of acute procedural success and symptom improvement. The relationship between ablation and other clinical outcomes has been limited to small studies primarily conducted at academic centers. We sought to determine if catheter ablation of atrial flutter is associated with reductions in healthcare utilization, atrial fibrillation, or stroke in a large, real world population. California Healthcare Cost and Utilization Project databases were used to identify patients undergoing atrial flutter ablation between 2005 and 2009. The adjusted association between atrial flutter ablation and healthcare utilization, atrial fibrillation, or stroke was investigated using Cox proportional hazards models. Among 33,004 patients with a diagnosis of atrial flutter observed for a median of 2.1 years, 2,733 (8.2%) underwent catheter ablation. Atrial flutter ablation significantly lowered the adjusted risk of inpatient hospitalization (HR 0.88, 95% CI 0.84–0.92, p<0.001), emergency department visits (HR 0.60, 95% CI 0.54–0.65, p<0.001), and overall hospital-based healthcare utilization (HR 0.94, 95% CI 0.90–0.98, p = 0.001). Atrial flutter ablation was also associated with a statistically significant 11% reduction in the adjusted hazard of atrial fibrillation (HR 0.89, 95% CI 0.81–0.97, p = 0.01). Risk of acute stroke was not significantly reduced after ablation (HR 1.09, 95% CI 0.81–1.45, p = 0.57). In a large, real world population, atrial flutter ablation was associated with significant reductions in hospital-based healthcare utilization and a reduced risk of atrial fibrillation. These findings support the early use of catheter ablation for the treatment of atrial flutter.

## Introduction

The efficacy of endocardial catheter ablation for the treatment of atrial flutter (AFL) is well established. AFL ablation is associated with a high rate of acute procedural success [1] and a low incidence of AFL recurrence during follow up [2]. In addition, randomized comparisons of ablation versus medical management have shown significantly less symptoms, reduced morbidity, and enhanced quality of life with an ablation strategy [3], [4].

The impact of AFL ablation on other arrhythmia related clinical outcomes, however, is less clear. Although previous investigations have found an association between AFL ablation and a reduction in subsequent healthcare visits, these small studies have been limited to single academic centers [5], [6] or to carefully selected randomized trial participants [3]. Furthermore, while one randomized trial demonstrated less atrial fibrillation (AF) after AFL ablation [3], this finding was not replicated in a second study [4]. Finally, although AFL ablation could potentially reduce the risk of thromboembolic stroke through maintenance of sinus rhythm, prior investigations have not been powered to assess for differences in this endpoint.

The relationship between AFL ablation and these important clinical outcomes has not been studied in a large, real world population. We therefore used data from the Healthcare Cost and Utilization Project (HCUP) to determine if catheter ablation is associated with reductions in healthcare utilization, AF, and stroke among a contemporary population of California residents diagnosed with AFL.

## Materials and Methods

### Ethics Statement

Patient information was anonymized prior to analysis and certification to use this deidentified HCUP data was obtained from the University of California, San Francisco Committee on Human Research.

We identified all patients ≥18 years of age with a diagnosis of AFL who received care in a California emergency department, inpatient hospital unit, or ambulatory surgery setting between January 1, 2005 and December 31, 2009 using HCUP (Agency for Healthcare Research and Quality) California State Emergency Department Databases, State Inpatient Databases, and State Ambulatory Surgery Databases [7]. Individual databases specific to calendar year and healthcare setting were merged using an encrypted linkage variable to identify repeat visits for a given patient. Patients with missing admission date data, residence outside of the state of California, or concomitant AF (defined as an AF diagnosis either before or at the same time as an AFL diagnosis) were excluded. For the healthcare utilization outcome, individuals entered the study cohort upon their first AFL diagnosis and were censored after inpatient death or at the end of the study period (December 31, 2009). For AF and stroke analyses, patients were additionally censored at the time of the respective outcome of interest.

Age, gender, race, income level, and insurance payer were recorded at each healthcare encounter by the discharging institution. Income level was categorized by quartiles using the median household income for the patient's ZIP code. Up to 25 International Classification of Diseases-9th Edition (ICD-9) codes and 21 Current Procedural Terminology (CPT) codes were provided for each encounter. AFL and AF were defined using the ICD-9 codes 427.32 and 427.31, respectively. Because post-operative AFL and AF may have a different underlying mechanism than when observed outside of the acute surgical setting, AFL and AF were not recorded if a patient had undergone cardiothoracic surgery during the same hospitalization or within the previous 30 days [8]. Other medical comorbidities postulated to confound and/or mediate the association between AFL ablation and clinical outcomes were also recorded using ICD-9 and CPT codes (**Table S1**) [8], [9]. Dichotomous medical comorbidity variables were accumulated at each healthcare encounter and carried forward over time. The Charlson Comorbidity Index was calculated at each discharge event using ICD-9 codes as previously described [10].

AFL ablation procedures were identified using the ICD-9 code for endocardial catheter ablation (37.34) in patients with a concomitant diagnosis of AFL. To maintain the specificity of AFL ablation identification, patients with a diagnosis of AF, supraventricular tachycardia, ventricular tachycardia, premature ventricular contractions, Wolf-Parkinson-White syndrome, Lown–Ganong–Levine syndrome, atrio-ventricular nodal reentrant tachycardia, or implantable pacemaker or defibrillator insertion coded at the same time of the ablation procedure were not considered to have undergone AFL ablation [9].

Healthcare utilization was defined as any inpatient hospitalization, emergency department visit without inpatient admission, or ambulatory surgery encounter. Acute ischemic stroke/transient ischemic attack and AF were identified using ICD-9 coding (**Table S1**).

### Statistical Analysis

Continuous variables with a normal distribution are presented as mean ± standard deviation (SD) and were compared using t-tests. Non-normally distributed continuous variables are presented as medians with interquartile ranges (IQR) and were compared using Kruskal-Wallis tests. The association between categorical variables was determined using Chi-squared tests. Cox proportional hazards models were used to investigate the association between AFL ablation and clinical outcomes both before and after controlling for known confounders identified *a priori*. In these models, AFL ablation, insurance payer, income level, and medical comorbidities were treated as time-dependent covariates. The proportional hazards assumption was assessed using Kaplan-Meier versus predicted survival plots and log-minus-log survival plots. For the assessment of healthcare utilization, a patient who underwent AFL ablation could contribute observation time and events to both the non-ablation and ablation groups depending upon ablation status. To reduce systematic bias in favor of ablation, the AFL ablation visit was considered a post ablation healthcare encounter. AF and stroke analyses were limited to individuals with a first healthcare encounter between 2006 and 2009 to ensure adequate exclusion of patients with prevalent AF and stroke, respectively.

Several sensitivity analyses were performed to further evaluate the observed association between ablation and healthcare utilization. First, a comparison of medical visits before and after ablation was restricted to only those patients who underwent AFL ablation. Such an analysis should minimize selection bias, as healthcare utilization was compared before and after ablation within individual patients. We also recognized that non-cardiac conditions likely impact both a provider's decision to perform AFL ablation and healthcare utilization. A second analysis therefore compared healthcare utilization by AFL ablation status after adjusting for patient demographics, cardiovascular risk factors, and Charlson Comorbidity Index. The Charlson Comorbidity Index is a validated instrument that uses the presence of a wide variety of medical conditions to estimate an individual patient's relative mortality [11]. This scoring system has been adapted to administrative databases that utilize ICD-9 coding [10] and serves as an overall estimate of a patient's health status. In addition, we utilized propensity score methods to address confounding between the ablation and non-ablation groups. For these analyses, a logistic model that included age, gender, race, insurance, income, hypertension, diabetes, coronary artery disease, heart failure, valvular heart disease, pulmonary disease, chronic kidney disease, and neurologic disease was used to estimate the probability of AFL ablation for each patient at the time of index AFL diagnosis. Propensity scores were treated as either a categorical variable (expressed as quintiles) or a continuous measurement (modeled using restricted cubic splines) and included in Cox proportional hazard models to determine the adjusted association between AFL ablation and healthcare utilization. Finally, we also performed a propensity score matched analysis whereby patients undergoing ablation were matched 1∶1 with non-ablated individuals using a nearest neighbor matching algorithm without replacement.

All analyses were performed using Stata 12 (StataCorp, College Station, TX, USA). A two-tailed p<0.05 was considered statistically significant.

## Results

**Figure 1:**
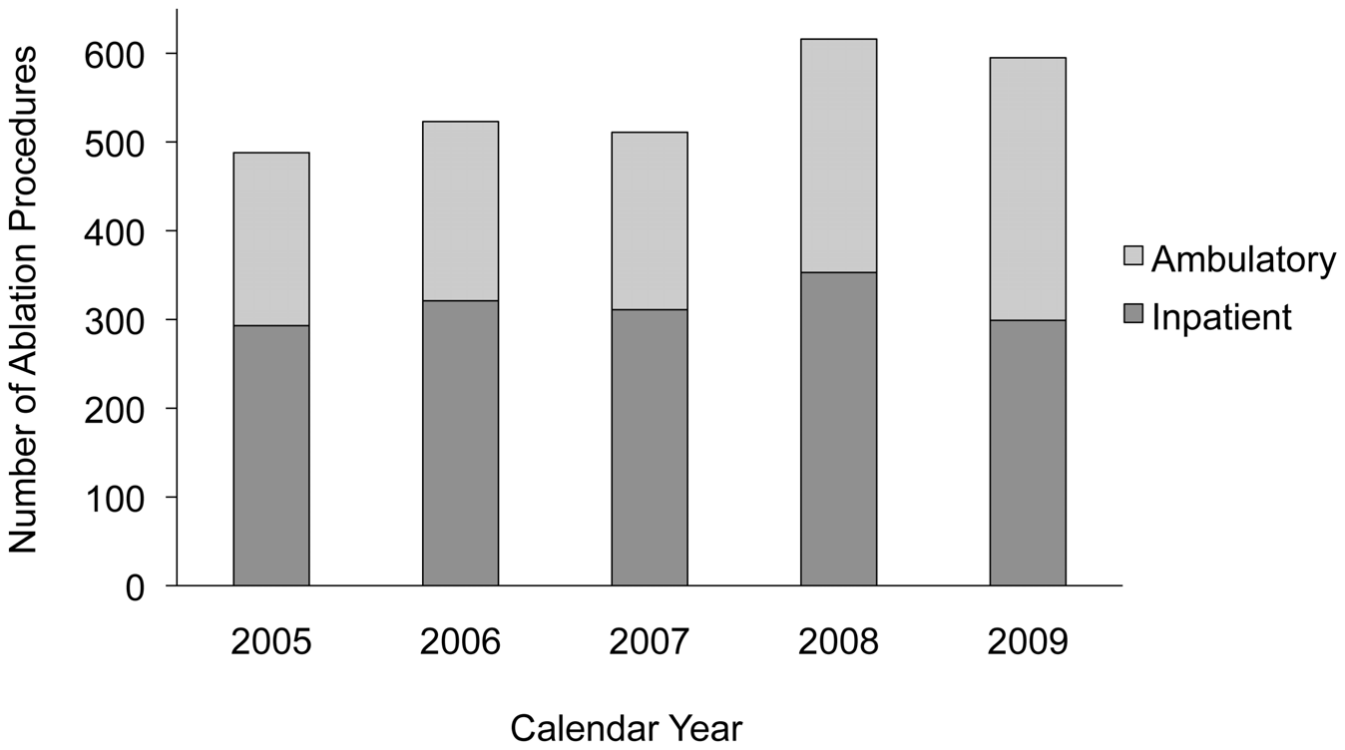
Atrial Flutter Ablation Procedures by Calendar Year and Healthcare Setting. The absolute number of ablation procedures performed in an ambulatory surgery (light bar) or inpatient hospitalization (dark bar) setting is shown for each calendar year included in the study.

Among 33,004 patients with a diagnosis of AFL observed for a median of 2.1 (IQR 0.8 to 3.6) years, 2,733 (8.2%) underwent catheter ablation. Ablation procedures were fairly evenly distributed over the study period and were roughly equally divided between inpatient and outpatient procedural settings (**Figure 1**). The ablated group was significantly younger and had a greater proportion of men, a lower prevalence of cardiovascular comorbidities, and a lower median Charlson Comorbidity Index (**Table 1**).

**Table 1:** Patient Demographics and Comorbidities at First Diagnosis of Atrial Flutter by Ablation Status.

	Ablated n = 2,733	Non-Ablated n = 30,271	P value
**Age, mean (SD), years**	65 (13)	70 (14)	<0.001
**Female, n (%)**	593 (22)	11,592 (38)	<0.001
**Insurance, n (%)**			<0.001
Medicare	1,614 (59)	21,670 (72)	
Medicaid	95 (3)	1,862 (6)	
Private	972 (36)	6,019 (20)	
Self-Pay	13 (1)	443 (1)	
Other	39 (1)	276 (1)	
**Income Quartile, n (%)**			<0.001
1 Poorest	441 (16)	5,476 (18)	
2	560 (21)	6,341 (21)	
3	749 (28)	8,665 (29)	
4 Wealthiest	951 (35)	9,476 (32)	
**Hypertension, n (%)**	1,211 (44)	17,292 (57)	<0.001
**Diabetes, n (%)**	494 (18)	8,418 (28)	<0.001
**Coronary Artery Disease, n (%)**	632 (23)	9,642 (32)	<0.001
**Heart Failure, n (%)**	465 (17)	8,861 (29)	<0.001
CTS*, n (%)	0	16 (0.1)	0.23
**Valvular Disease, n (%)**	363 (13)	4,265 (14)	0.24
**Pulmonary Disease, n (%)**	285 (10)	6,481 (21)	<0.001
**Chronic Kidney Disease, n (%)**	111 (4)	3,595 (12)	<0.001
**Charlson Comorbidity Index, median (IQR)**	0 (0 to 1)	1 (0 to 2)	<0.001

CTS, cardiothoracic surgery; IQR, interquartile range; SD, standard deviation.

### AFL Ablation and Healthcare Utilization

**Figure 2:**
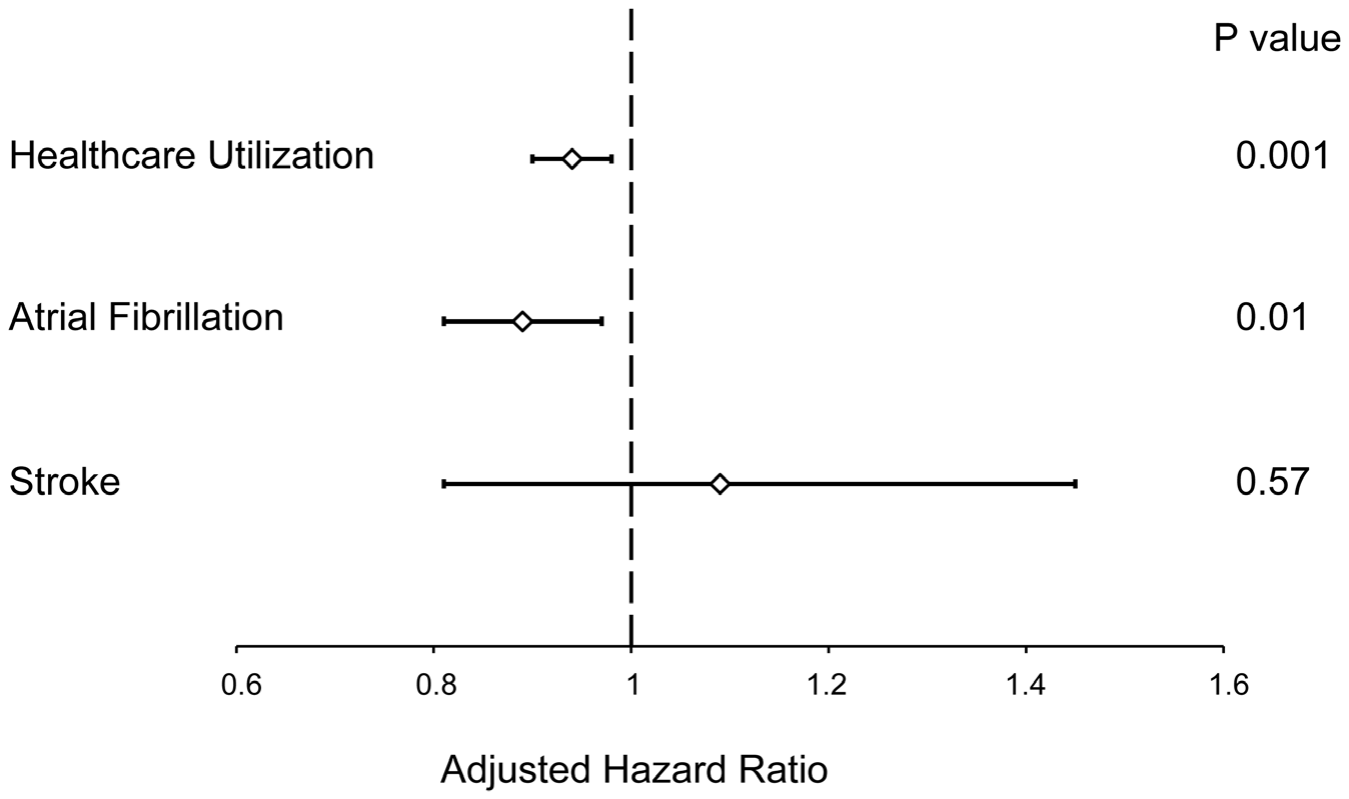
Adjusted Hazard of Healthcare Utilization, Atrial Fibrillation, or Stroke After Atrial Flutter Ablation. Diamonds indicate the adjusted hazard ratio point estimates and error bars denote 95% confidence intervals. The dashed vertical line represents a hazard ratio of 1 (no difference with atrial flutter ablation).

A total of 135,614 healthcare encounters were observed among all atrial flutter patients with a median of 3 (IQR 2 to 6) visits per patient. When not ablated, there were 1.86 visits per person-year (95% CI 1.85 to 1.87). After ablation, there were 1.50 visits per person-year (95% CI 1.47 to 1.53). In multivariate analysis adjusting for patient demographics (age, gender, race, insurance, and income) and comorbidities (hypertension, diabetes, coronary artery disease, heart failure, remote history of cardiothoracic surgery, valvular heart disease, pulmonary disease, chronic kidney disease, neurologic disease, and atrial fibrillation), AFL ablation resulted in a significantly increased hazard of an ambulatory surgery encounter (HR 1.63, 95% CI 1.54 to 1.73, p<0.001). However, ablation significantly lowered the risk of inpatient hospitalization by 12% (HR 0.88, 95% CI 0.84 to 0.92, p<0.001) and cut the risk of an emergency department visit by 40% (HR 0.60, 95% CI 0.54 to 0.65, p<0.001, **Table 2**). This resulted in a statistically significant reduction in the adjusted hazard of *overall* healthcare utilization (including ambulatory surgery encounters, inpatient hospitalizations, and emergency department visits) with AFL ablation (HR 0.94, 95% CI 0.90 to 0.98, p = 0.001, **Figure 2**).

**Table 2:** Adjusted Hazard of Healthcare Utilization by Setting.

Healthcare Setting	Adjusted HR*	95% CI	P value
Ambulatory Surgery	1.63	1.54 to 1.73	<0.001
Inpatient Hospitalization	0.88	0.84 to 0.92	<0.001
Emergency Department Visit	0.60	0.54 to 0.65	<0.001
Overall Healthcare Utilization	0.94	0.90 to 0.98	0.001

Adjusted for age, gender, race, insurance, income, hypertension, diabetes, coronary artery disease, heart failure, remote history of cardiothoracic surgery, valvular heart disease, pulmonary disease, chronic kidney disease, neurologic disease, and atrial fibrillation. Overall healthcare utilization includes ambulatory surgery, inpatient, and emergency department encounters. CI, confidence interval; HR, hazard ratio.

In a sensitivity analysis controlling for the Charlson Comorbidity Index in addition to the above demographic and comorbidity variables, the hazard of healthcare utilization remained significantly reduced with ablation (HR 0.93, 95% CI 0.89 to 0.96, p<0.001). When analysis was limited to only those patients who underwent catheter ablation (comparing visits before versus after ablation), AFL ablation was associated with a near 50% reduction in the adjusted hazard of overall healthcare utilization (HR 0.51, 95% CI 0.47 to 0.55, p<0.001). In analyses treating the AFL ablation propensity score as continuous predictor, ablation was again found to be associated with a reduction in the hazard of overall healthcare utilization (HR 0.91, 0.87 to 0.95, p<0.001). This result did not substantially differ when the propensity score was modeled as a categorical variable. Similarly, in a 1∶1 propensity matched analysis, AFL ablation remained associated with a significant reduction in overall healthcare utilization (HR 0.86, 0.81 to 0.90, p<0.001).

### AFL Ablation and AF

From the population of AFL patients without a known diagnosis of AF, we observed 11,237 incident episodes of AF. When not ablated, the rate of incident atrial fibrillation was 23.0 per one hundred person years (95% CI 22.5 to 23.6). After flutter ablation, the rate was 16.9 per one hundred person years (95% CI 15.5 to 18.4). In multivariate analysis adjusting for age, gender, race, income, insurance status, and history of hypertension, diabetes, coronary artery disease, heart failure, cardiac surgery, valve disease, pulmonary disease, and chronic kidney disease, AFL ablation was associated with a statistically significant 11% reduction in the hazard of AF (HR 0.89, 95% CI 0.81 to 0.97, p = 0.01, **Figure 2**).

### AFL Ablation and Stroke

We observed 1,203 incident acute strokes during the study period. When not ablated, the rate of incident stroke was 17.9 per thousand person years (95% CI 16.7 to 19.3). After flutter ablation, the rate was 13.1 per thousand person years (95% CI 10.0 to 17.3). In multivariate analysis adjusting for age, gender, race, insurance, income, and history of AF, hypertension, diabetes, coronary disease, heart failure, cardiac surgery, valvular heart disease, and chronic kidney disease, AFL ablation was not significantly associated with acute stroke (HR 1.09, 95% CI 0.81 to 1.45, p = 0.57, **Figure 2**).

## Discussion

In a large, real world population of patients diagnosed with AFL, we found that AFL ablation significantly reduced overall healthcare utilization. The lower rate of healthcare encounters after ablation was driven by substantial reductions in all-cause inpatient hospitalization and emergency department visits. AFL ablation was also associated with a reduced incidence of post-procedural AF, although ablation did not reduce the hazard of acute stroke.

Prior observational studies and randomized trials have demonstrated decreased healthcare visits after AFL ablation [3], [5], [6]. Extrapolation of these previous investigations to the broad population of patients with AFL, however, is limited due to their small sample size (generally 100 or less patients), use of patient recall to identify hospitalization encounters, or selective enrollment in the context of a clinical trial. In addition, many of the studies examining this association have been performed at academic centers outside of the United States, where alternative healthcare cost and delivery forces may influence hospital utilization [5], [6]. Our findings extend this prior research by demonstrating a reduction in objectively measured hospital-based encounters among real world patients treated at both community and academic hospitals across California.

We found that AFL ablation reduced the risk of overall healthcare utilization by 6%. It is important to note that our primary outcome included *all* hospital-related healthcare encounters before and after ablation. This analysis strategy was utilized to provide a conservative estimate of AFL ablation benefit, as the ablation procedure would only be expected to reduce AFL-related admissions. The ambulatory surgery encounter during which a patient underwent AFL ablation was counted as a post-ablation visit, likely accounting for the significantly increased hazard of an ambulatory surgery visit in the ablation group. Notably, AFL ablation sufficiently reduced subsequent inpatient hospitalizations and emergency department visits to offset the increased encounters incurred by the procedure itself.

Multiple sensitivity analyses were utilized to further explore the observed association between AFL ablation and healthcare outcomes. As we reasoned that a patient's overall health status would likely influence the likelihood of AFL ablation, we further adjusted our primary analysis for the Charlson Comorbidity Index. This metric was previously developed to estimate an individual patient's relative mortality using medical comorbidities identified from ICD-9 coding [10], [11]. A separate analysis quantified hospital-based healthcare encounters only in patients who underwent ablation. As overall healthcare utilization was significantly decreased within individual patients after the ablation procedure, these results strongly argue against residual confounding as an explanation for our overall findings. Finally, we utilized both propensity score adjustment and matching as an additional and complementary methodology to minimize bias. Our sensitivity analyses consistently demonstrated a reduction in healthcare utilization with AFL ablation, strengthening the results of the primary analysis. The use of catheter ablation to treat a first episode of AFL is currently a Class IIa recommendation; this procedure only receives a Class I recommendation after arrhythmia recurrence [12]. Given the overall efficacy and safety of this procedure for the treatment of AFL, our findings may support the use of catheter ablation as a first-line treatment for AFL.

Although the electrophysiologic mechanisms of AFL and AF are distinct, the two arrhythmias often coexist and may share a common trigger [13]–[15]. Randomized trials comparing AFL ablation to medical therapy have reached divergent results, with one trial demonstrating a reduced risk of AF post-ablation [3] and a second showing no difference in AF risk [4]. Our results suggest AF risk is modestly but significantly decreased after AFL ablation, supporting the hypothesis that AFL ablation can reduce pathologic atrial changes that increase AF incidence. To our knowledge, this is the first investigation that has compared long-term stroke outcomes before and after AFL ablation. As AFL is known to increase the risk of cardiogenic thromboembolism, we hypothesized that ablation of this arrhythmia could reduce stroke incidence. The observed hazard ratio between the ablated and non-ablated groups, however, was small and did not reach statistical significance. Our findings are in agreement with a recent study from a single, experienced academic center that reported substantial AF and stroke risk after AFL ablation [16]. This prior investigation, however, had comparatively few events and used historic controls to identify the heightened risk of these post-ablation outcomes. Our comparison of events among patients that did and did not receive an AFL ablation in a large, multicenter population extends these findings and establishes a population-based relative risk of AF and stroke outcomes after ablation.

Limitations of the present study should be recognized. Outcome and confounder variables were determined using hospital discharge coding and residual confounding cannot be excluded. Nevertheless, we believe this is less likely to explain our healthcare utilization results given that our findings persisted after sensitivity analyses. HCUP databases do not include ambulatory clinic encounters and we were therefore not able to compare such visits in our analysis. However, the most costly healthcare visits (emergency department and inpatient hospitalizations) were optimally captured by the HCUP database. We similarly did not have information regarding the use of anticoagulant or antiarrhythmic medications, which could have implications for our AF and stroke outcomes. For example, it possible that AFL ablation patients were more likely to receive care from an electrophysiologist, who may be more likely to prescribe an antiarrhythmic drug. In addition, the methodology used for AFL ablation identification in our analysis was developed to favor specificity over sensitivity. To our knowledge, there are no large, contemporary, population-based samples that define the overall proportion of AFL patients treated with catheter ablation. As such, the degree to which we have underestimated overall ablation utilization cannot be accurately quantified and the absolute rate at which this procedure is utilized in real world settings cannot be directly extrapolated from our analysis. Given the de-identified nature of the HCUP dataset, it was not possible to validate the accuracy of ICD-9 coding for AFL and AF. Notably, to bias our estimation of the association between AFL ablation and AF, the rate of misdiagnosis of these arrhythmias would need to differ by history of ablation. Because such a scenario is unlikely, we believe that widespread differential misclassification of AF and AFL by ablation status is unlikely to explain our positive results. Although we excluded arrhythmia episodes that occurred immediately after cardiac surgery, we were unable to determine which flutter diagnoses occurred in the setting of other reversible triggers (such as pneumonia or hyperthyroidism) and therefore might not be appropriately treated with an ablation procedure. Finally, because we did not have information regarding the flutter mechanism, the proportion of patients with a typical, cavotricuspid isthmus dependent arrhythmia circuit is not known (although approximately 90% of clinically observed flutter circuits are thought to involve this mechanism) [17].

In conclusion, we observed significant reductions in healthcare utilization after catheter ablation among patients with AFL. In addition, catheter ablation was associated with a reduced risk of AF. These findings support the early use of catheter ablation in the treatment of AFL.

## Supporting Information

Table S1 **International Classification of Diseases-9th Edition (ICD-9) and Current Procedural Terminology (CPT) Codes Used for Disease Identification.**
(DOCX)
