# Supplementary material for: Healthcare Utilization and Clinical Outcomes after Catheter Ablation of Atrial Flutter
Source: PLoS One. 2014 Jul 1;9(7):e100509. doi: 10.1371/journal.pone.0100509 (PMC4077565; doi:10.1371/journal.pone.0100509)
Supplement: Table S1 — International Classification of Diseases-9th Edition (ICD-9) and Current Procedural Terminology (CPT) Codes Used for Disease Identification. (DOCX) [file pone.0100509.s001.docx]

**Supplemental Table S1** International Classification of Diseases-9th Edition (ICD-9) and Current Procedural Terminology (CPT) Codes Used for Disease Identification

| **Diagnosis** | **ICD-9 / CPT Codes** | |
| --- | --- | --- |
|  |  |  |
| Atrial Flutter | ICD-9 | 427.32 |
| Atrial Fibrillation | ICD-9 | 427.31 |
| Acute Ischemic Stroke/TIA | ICD-9 | 433.01, 433.11, 433.21, 433.31, 433.81, 433.91, 434.01, 434.11, 434.91, 435.8, 435.9 |
| Hypertension | ICD-9 | 401.X, 402.X, 403.X, 404.X, 405.X, 437.2 |
| Diabetes | ICD-9 | 249.X, 250.X, 790.X, 791.5, 791.6, V458.5, V539.1, V654.6 |
| Coronary Artery Disease | ICD-9 | 36.01, 36.02, 36.03, 36.05, 36.09, 36.1X, 411.0,  411.1, 411.8, 411.89, 412, 413.X, 414.X, 429.7, V458.2 |
| Heart Failure | ICD-9 | 402.01, 402.11, 402.91, 404.91, 404.93, 425,X, 428.X |
| Cardiothoracic Surgery* | ICD-9 | 35.3X, 35.41, 35.42, 35.50, 35.51, 35.52, 35.53, 35.54, 35.60, 35.61, 35.62, 35.63, 35.70, 35.71, 35.72, 35.73 36.1X, 37.10, 37.11, 37.12, 37.24, 37.25, 37.31, 37.32, 37.33, 37.35, 37.40 |
| Valvular Disease | ICD-9 | 394.X, 395.X, 396.X, 397.0, 397.1, 424.0, 424.1, 424.2, 424.3, V422, V433 |
| Pulmonary Disease | ICD-9 | 494.2X, 491.8, 491.9, 492.0, 492.8, 494, 494.0, 494.1, 496 |
| Chronic Kidney Disease | ICD-9  CPT | 39.93, 54.98, 585.X, V420, V451, V451.1, V451.2, V560, V561, V562, V563.1, V563.2, V568, V56  90921, 90925, 90935, 90937, 90945, 90947, 90989, 90993 |
| Neurologic Disease | ICD-9 | 430, 431, 432.X, 433.X, 434.X, 435.X, 438.X |

*Atrial fibrillation was blanked if diagnosed within 30 days after cardiothoracic surgery. TIA, transient ischemic attack.
